# The effect of various kinematics on postoperative pain after instrumentation: a prospective, randomized clinical study

**DOI:** 10.1590/1678-775720160136

**Published:** 2016

**Authors:** Hakan Arslan, Ruslan Khalilov, Ezgi Doğanay, Ertugrul Karatas

**Affiliations:** Ataturk University, Faculty of Dentistry, Department of Endodontics, Erzurum, Turkey.

**Keywords:** Apically extruded debris, Reciproc, Motion, Endodontic treatment, Postoperative pain

## Abstract

**Objective:**

To evaluate various kinematic movements on postoperative pain using a Reciproc system.

**Material and Methods:**

Fifty-six molar teeth were divided into four groups according to kinematics as follows: continuous rotation, 360° CCW – 30° CW, 270° CCW – 30° CW, and 150° CCW – 30° CW. Preoperative and postoperative pain levels using visual analogue scale (VAS), percussion pain, and analgesic intake were recorded for each subject. Postoperative pain levels at 1, 3, 5, and 7 d were evaluated. Data were analyzed statistically using the Kruskal-Walis, Mann-Whitney-U, one-way analysis of variance, and chi-square tests (p=0.05).

**Results:**

Continuous rotation resulted in more pain at Day 1 when compared with the reciprocating groups (360° CCW – 30° CW and 270° CCW – 30° C) (p<0.05).

**Conclusions:**

Continuous rotation resulted in more postoperative pain at Day 1 than in reciprocating groups, and thereafter no significant pain was found among the groups.

## INTRODUCTION

One of the most important matters in endodontic treatment is the prevention of pain. Postoperative pain after endodontic treatment is a frequent complication. According to a systematic review, the frequency of endodontic postoperative pain in patients is between 3% and 58%^11^. Postoperative pain can be affected by almost all of the procedures in root canal treatment, including anaesthesia administration^6^, introduction of glide path^8^, use of instrumentation systems^4,5^, and retreatment^13^.

Reciproc system (VDW, Munich, Germany) is characterized by an S-shaped cross section. It has sharp cutting edges and a non-cutting tip. It shapes the canals with a reciprocal back-and-forward motion (150 degrees counterclockwise and then 30 degrees clockwise). This single file-system consists of three files; R25 (25/0.08), R40 (40/0.06), and R50 (50/0.05)^2^. These instruments are produced with a special NiTi alloy (M wire) subjected to a special thermal treatment process, performed to provide higher flexibility to the instrument^9^.

Previous studies have reported conflicting results on postoperative pain regarding the effect of instrumentation using reciprocating and rotation^4,5^. Neelakantan and Sharma^4^ (2015) evaluated postoperative pain after instrumentation of root canals with a single-file reciprocating (Reciproc) and rotary (One Shape, MicroMega, France) file systems, concluding that Reciproc showed significantly less intensity and duration of postoperative pain than One Shape. However, Nekoofar, et al.^5^ (2015) compared the intensity and duration of postoperative pain using WaveOne and ProTaper Universal, systems for root canal instrumentation, and found that postoperative pain was significantly lower in patients undergoing canal instrumentation with ProTaper Universal rotary instruments than with the WaveOne reciprocating single-file technique. These conflicting results could be a result of the use of instruments with different designs and/or the number of instruments used. We believe that there is nothing about the effect of different kinematics using the same instruments on postoperative pain in the literature. Therefore, the purpose of this study was to evaluate four (combinations of) kinematic movements [counter clockwise (CCW) continuous rotation, 360° CCW −30° clockwise (CW), 270° CCW – 30° CW, and 150° CCW – 30° CW] regarding postoperative pain using just one type of instrument, a Reciproc system. The null hypothesis was that there is no difference among the groups in postoperative pain.

## MATERIAL AND METHODS

**Figure 1:**
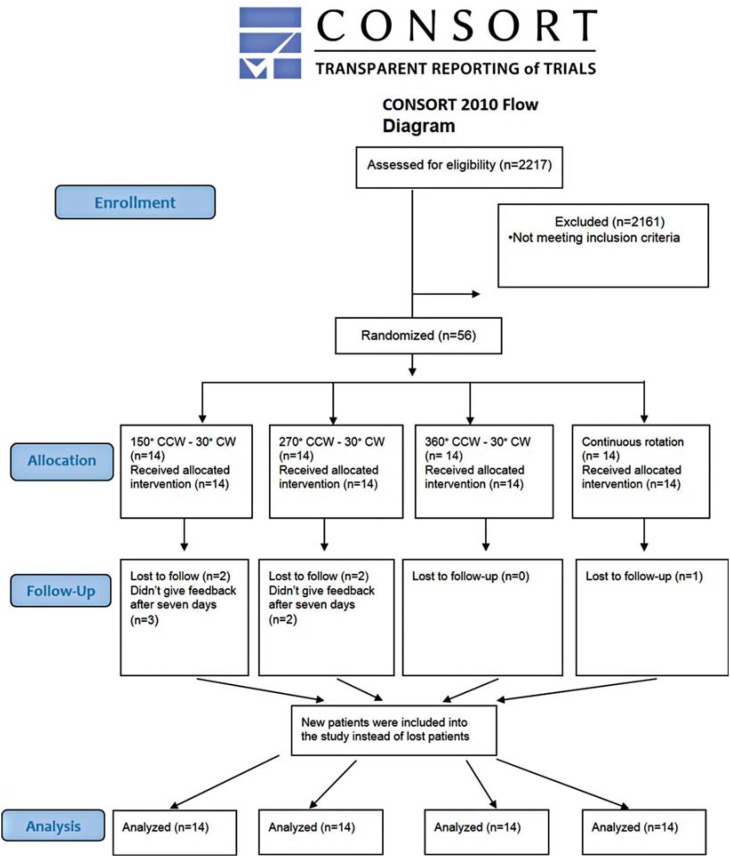
Consort flow diagram

The protocol was approved by the Research Ethics Committee. Sample size was calculated as 56 with a power of 0.80 (effect size=0.46). A total of 56 patients were selected for this *in vivo* study. Study subjects were recruited from the pool of patients referred to the Department of Endodontics for root canal treatment from May 2015 to October 2015 (6 months). Figure 1 shows the consort flow diagram.

### Inclusion criteria

1. Healthy patients without any systematic diseases or allergic reactions;
2. Tooth responsive to cold test;
3. Patients with maxillary and mandibular molars;
4. Patients with a preoperative pain level from 0 to 25 on the visual analogue scale (VAS) of 100 mm length.

### Exclusion criteria

1. Palpation pain;
2. Bruxism or clenching;
3. Antibiotics or analgesics taken in the past 24 h;
4. Previous root canal treatment;
5. Swelling or sinus tract;
6. Severe periodontal disease;
7. Pocket depth greater than 5 mm;
8. Mobility greater than grade 1;
9. Periapical radiolucency;
10. Severely damaged teeth;
11. Absence of occlusal contact;
12. Treatment with technical difficulty;
13. Teeth with problems, such as over-instrumentation, broken files, and problems in determining working length.

The patients were randomly distributed into the groups using a web program (available at www.randomizer.org). Patient and group numbers were recorded on paper. After each patient signed the informed consent form, the tooth was anesthetized with a local anaesthetic solution containing 1.7 mL of 4% articaine with 1:100 000 epinephrine (UltracaineDS^®^ forte; Aventis, Istanbul, Turkey). A buccal infiltration anaesthesia and palatal injection were performed. The procedure was initiated 15 min later.

After a straight-line access cavity was prepared, the procedure was completed under rubber-dam isolation. The working length was determined by an electronic apex locator (Root ZX mini; J. Morita Mfg Corp., Kyoto, Japan), and the Reciproc instruments were used according to the manufacturer's instructions. A new Reciproc instrument was used for each patient. Palatine canals of maxillary molars and distal canals of mandibular molars were finished with R40, and the others were finished with R25. The patients were divided into four groups (n=14) according to the kinematic movements, as follows: counterclockwise continuous rotation, 360° CCW – 30° CW, 270° CCW – 30° CW, and 150° CCW −30° CW.

For all groups, the speed of the motor was adjusted to 300 rpm. For the continuous rotation group, 200 g/cm torque was used. A size 10 K-file was used to maintain apical patency. For the irrigation, 2 mL of 1.25% NaOCl was used between in-and-out pecking motions with safety tip needle (Canal Clean; Biodent, Paju, Korea) approximately 4 mm short from working length, and a final rinse was performed using 1.25% NaOCl and 17% EDTA for 1 min to remove the smear layer.

After root canal preparation, the root canals were dried with paper points and then filled using matched single cones and AH Plus sealer (Dentsply De Trey GmbH, Konstanz, Germany). The pulp chamber was filled with a flowable composite resin, and a nanohybrid composite resin was inserted into the cavity using an incremental technique and cured for 20 s using a LED light-curing unit (Valo Cordless, Ultradent, South Jordan, UT, USA) with an output of 1000 mW/cm^2^.

The patients were instructed to use 400 mg ibuprofen (Artril; Eczacıbaşı, Istanbul, Turkey) if the pain was bearable and informed to record the analgesic intake on a customized form, which was also used by them to record any pain experience. The following variables were recorded:

Age;
Gender;
Tooth number;
Preoperative pain on the VAS;
Preoperative and postoperative percussion pain levels on the VAS;
Pain level on the 1^st^, 3^rd^, 5^th^, and 7^th^ days;
Analgesic intake after the procedure.
Change in pain was calculated at the related day based on the preoperative pain.

### Statistical analysis

The Kolmogorov-Smirnov test for distribution of data for reduction in pain levels, according to the day, revealed non-normal distribution. Thus, data were analyzed using the Kruskal-Walis and Mann-Whitney-U tests for inter group analysis (p=0.05). The differences in age and preoperative and postoperative percussion pain levels were statistically analyzed using one-way ANOVA test (p=0.05). The differences in gender and analgesic intake were statistically analyzed using a chi-square test (p=0.05).

## RESULTS

Table 1 shows the demographic data related to age, gender, preoperative and postoperative percussion, palpation, swelling, and sinus tract.

**Table 1:** Demographic data (One-way Anova and chi-square were used to analyze the data) (SD: standard deviation)

	150° CCW − 30° CW	270° CCW − 30° CW,	360° CCW − 30° CW	Continuous rotation	p value
Age	36.36±12.46	27.07±12.71	29.07±11.77	31.79±9.93	.193
Gender (Female*Male)	5*9	9*5	5*9	7*7	.368
Mean ± SD VAS value of preoperative pain	3.57±9.07	1.79±5.40	1.07±4.00	0.00±0.00	.406
Mean ± SD VAS value of preoperative percussion pain	15.57±24.85	5.86±13.10	9.64±12.98	11.00±12.96	.209
Numbers of patients with preoperative palpation sensitivity	0	0	0	0	–
Numbers of patients with preoperative swelling	0	0	0	0	–
Numbers of patients with preoperative sinus tract	0	0	0	0	–
Number of patients who intake preoperative drugs	0	0	0	0	–
Numbers of patients with necrotic pulp	0	0	1	0	.383
Numbers of patients with periapical lesion	0	0	0	1	.383
Numbers of patients who needed analgesics postoperatively	2	1	2	3	.761
Mean ± SD VAS value of postoperative percussion pain	3.71±7.44	0.00±0.00	0.00±0.00	3.64±10.30	.209
Numbers of patients with postoperative palpation sensitivity	0	0	0	0	–
Numbers of patients with postoperative swelling	0	0	0	0	–
Numbers of patients with postoperative sinus tract	0	0	0	0	–
Numbers of patients referred for an unscheduled appointment	0	0	0	0	–

**Figure 2:**
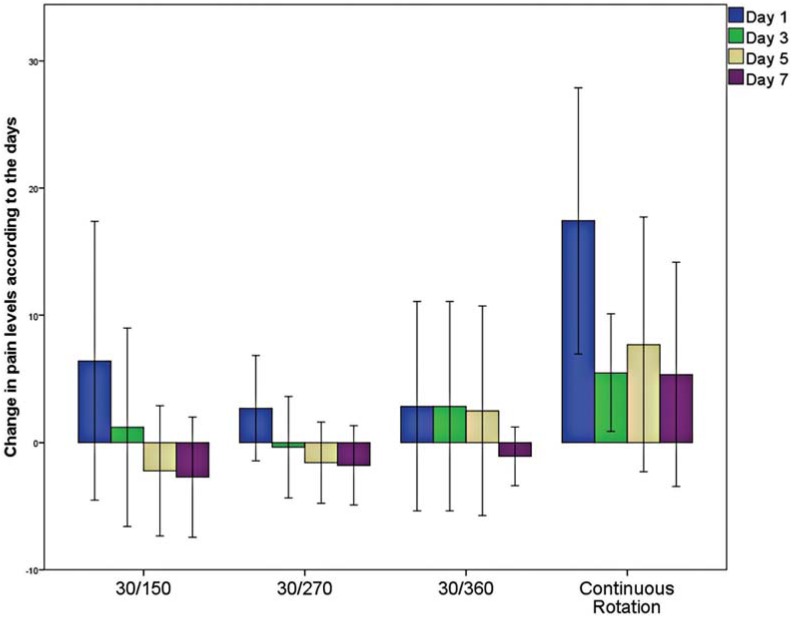
Change in pain levels according to the days. Change in pain was calculated at the day based on the preoperative pain. Continuous rotation increased pain at Day 1 in comparison with other reciprocating groups (360° CCW – 30° CW and 270° CCW – 30° C)

Figure 2 shows the reduction in pain levels at different time intervals. The Kruskal-Walis test revealed significant differences among the groups at Day 1 (p<0.05), but not at the other time periods (p>0.05). Mann-Whitney U test revealed that continuous rotation resulted in more pain at Day 1 than in reciprocating groups (360° CCW – 30° CW and 270° CCW – 30° C) (p<0.05).

The preoperative and postoperative percussion pain levels among the groups seven days after treatment were not statistically different (p>0.05).

None of the patients were referred to the clinic with swelling or sinus track after the treatment. Also, none of the patients needed an unscheduled appointment. Three patients in the continuous rotation group, two patients in the 360° CCW – 30° CW group, one patient in the 270° CCW – 30° CW, and two patients in the 150° CCW – 30° CW group had taken analgesics, postoperatively. No significant differences were found in analgesic use among the groups (p=0.761).

## DISCUSSION

Recently, Neelakantan and Sharma^4^ (2015) and Nekoofar, et al.^5^ (2015) evaluated postoperative pain after instrumentation of root canals with a single-file reciprocating or rotary file systems. However, one may argue that the different cross-sections, speed, kinematics, and systems may have had an impact on the postoperative pain. It is necessary, therefore, to evaluate the effect of pure reciprocating or continuous rotary motions using instruments with the same cross-sections on postoperative pain. Thus, the purpose of this study was to evaluate various kinematic movements on postoperative pain using just one type of instrument, a Reciproc system.

According to the results of this study, significant differences were found among the groups at Day 1 (p<0.05), but not at the other time periods (p>0.05) Thus, the null hypothesis was partially rejected.

An interesting finding was that continuous rotation resulted in more pain at Day 1 than in the reciprocating groups. Because there is no similar study in the literature, this finding cannot be compared with those of previous studies. Nekoofar, et al.^5^ (2015) compared the intensity and duration of postoperative pain using WaveOne and ProTaper Universal systems, for instrumentation of root canals, and found that postoperative pain was significantly lower in the patients undergoing canal instrumentation with ProTaper Universal rotary instruments. Pasqualini, et al.^7^ (2015) evaluated the impact of rotary and reciprocating instrumentation on postoperative quality of life and concluded that reciprocating instrumentation affected postoperative quality of life to a greater extent than rotary instrumentation. Relvas, et al.^10^ (2015) and Kherlakian, et al.^3^ (2016) did not find significant difference between different reciprocating and rotary systems in terms of postoperative pain. These findings are not in concordance with our results. However, a recent report by Neelakantan and Sharma^4^ (2015) concluded that Reciproc showed significantly less intensity and duration of postoperative pain compared with One Shape. Shokraneh, et al.^12^ (2016) evaluated postoperative pain after using hand files, ProTaper Universal, and Wave-One instruments and concluded that postoperative pain was significantly lower in Wave-One group. The results of the latter two studies corroborate our results.

A laboratory study by Arslan, et al.^1^ (2015) on the amount of apically extruded debris, using different kinematics, also supports our findings. According to the results of the study by Arslan, et al.^1^ (2015), the 150° CCW – 30° CW and 270° CCW – 30° CW reciprocating motions extruded significantly less debris than continuous rotation (p<0.05). In this study, the continuous rotation resulted in more pain at Day 1 than in reciprocating groups (360° CCW – 30° CW and 270° CCW – 30° C). Although the 150° CCW – 30° CW reciprocating motions extruded significantly less debris than continuous rotation in the laboratory study, in this study no significant difference was found between the 150° CCW – 30° CW reciprocating motions and continuous rotation. There are several explanations for the differences in the results of the studies, the most likely being the different methodologies *(in vivo* and *in vitro)* employed.

## CONCLUSION

Within the limitations of this study, continuous rotation resulted in more postoperative pain at Day 1 than in reciprocating groups (360° CCW – 30° CW and 270° CCW – 30° C), and, thereafter, no significant pain was found among the groups.

## References

[1] Arslan H, Doğanay E, Alsancak M, Çapar ID, Karataş E, Gündüz HA (2015). Comparison of apically extruded debris after root canal instrumentation using Reciproc® instruments with various kinematics. Int Endod J..

[2] Bane K, Faye B, Sarr M, Niang SO, Ndiaye D, Machtou P (2015). Root canal shaping by single-file systems and rotary instruments: a laboratory study. Iran Endod J..

[3] Kherlakian D, Cunha RS, Ehrhardt IC, Zuolo ML, Kishen A, Silveira Bueno CE (2016). Comparison of the incidence of postoperative pain after using 2 reciprocating systems and a continuous rotary system: a prospective randomized clinical trial. J Endod..

[4] Neelakantan P, Sharma S (2015). Pain after single-visit root canal treatment with two single-file systems based on different kinematics - a prospective randomized multicenter clinical study. Clin Oral Investig..

[5] Nekoofar MH, Sheykhrezae MS, Meraji N, Jamee A, Shirvani A, Jamee J (2015). Comparison of the effect of root canal preparation by using WaveOne and ProTaper on postoperative pain: a randomized clinical trial. J Endod..

[6] Parirokh M, Yosefi MH, Nakhaee N, Manochehrifar H, Abbott PV, Reza Forghani F (2012). Effect of bupivacaine on postoperative pain for inferior alveolar nerve block anesthesia after single-visit root canal treatment in teeth with irreversible pulpitis. J Endod..

[7] Pasqualini D, Corbella S, Alovisi M, Taschieri S, Del Fabbro M, Migliaretti G (2015). Postoperative quality of life following single-visit root canal treatment performed by rotary or reciprocating instrumentation: a randomized clinical trial. Int Endod J..

[8] Pasqualini D, Mollo L, Scotti N, Cantatore G, Castellucci A, Migliaretti G (2012). Postoperative pain after manual and mechanical glide path: a randomized clinical trial. J Endod..

[9] Pereira ES, Gomes RO, Leroy AM, Singh R, Peters OA, Bahia MG (2013). Mechanical behavior of M-Wire and conventional NiTi wire used to manufacture rotary endodontic instruments. Dent Mater.

[10] Relvas JB, Bastos MM, Marques AA, Garrido AD, Sponchiado EC (2015). Assessment of postoperative pain after reciprocating or rotary NiTi instrumentation of root canals: a randomized, controlled clinical trial. Clin Oral Investig.

[11] Sathorn C, Parashos P, Messer H (2008). The prevalence of postoperative pain and flare-up in single- and multiple-visit endodontic treatment: a systematic review. Int Endod J..

[12] Shokraneh A, Ajami M, Farhadi N, Hosseini M, Rohani B (2016). Postoperative endodontic pain of three different instrumentation techniques in asymptomatic necrotic mandibular molars with periapical lesion: a prospective, randomized, double-blind clinical trial. Clin Oral Investig.

[13] Yoldas O, Topuz A, Isçi AS, Oztunc H (2004). Postoperative pain after endodontic retreatment: single- versus two-visit treatment. Oral Surg Oral Med Oral Pathol Oral Radiol Endod..

